# Adopting U = U to end stigma and discrimination

**DOI:** 10.1002/jia2.25891

**Published:** 2022-02-28

**Authors:** Olivia G. Ford, Tinashe G. Rufurwadzo, Bruce Richman, Ian Green, Jacquelyne Alesi

**Affiliations:** ^1^ The Well Project Brooklyn New York USA; ^2^ Global Network of Young People Living with HIV Amsterdam Netherlands; ^3^ Prevention Access Campaign Washington DC USA; ^4^ Terrence Higgins Trust London UK; ^5^ Prevention Access Campaign Kampala Uganda

1

On Zero Discrimination Day, it is crucial to recommit to full adoption and advancement of Undetectable = Untransmittable (U = U) as a fundamental approach to ending stigma and discrimination against people living with HIV (PLHIV). Activists and researchers united in 2016 to launch U = U: building a scientific consensus and movement promoting the fact that when consistent adherence to HIV treatment suppresses the viral load of PLHIV to undetectable levels, there is no risk of onward transmission to sexual partners [1, 2].

The U = U message is well‐known within HIV research and advocacy communities. However, this can obscure the reality that U = U has not reached most PLHIV, nor most of the general population worldwide. Many community‐based and country‐wide efforts are underway towards integrating U = U into policy, communications and clinical practice. Yet, considerable work remains to use this message widely towards ending stigma and thus advancing health equity for PLHIV. In sub‐Saharan Africa, for instance U = U awareness remains very low and varies by HIV status [3]. Even in areas with higher levels of U = U awareness in some communities (such as in Europe, Asia, the Americas and Oceania), misinformation abounds regarding its effectiveness [3], limiting full realization of this powerful health strategy [4].

The potential impact of fully adopting U = U, both for PLHIV and the broader public, cannot be overstated. Knowledge and understanding of U = U can be a highly effective tool for destigmatizing HIV among the general population and reducing associated discrimination [5]. This is important because the negative effects of stigma and discrimination on the health and wellbeing of PLHIV are profound and well‐documented, including higher rates of depression and suicidal ideation, as well as lower social support, lower levels of adherence to antiretroviral treatment and decreased use of health and social services [6]. Experienced, as well as anticipated, stigma have been demonstrated to lower demand for HIV testing [7] and depress viral suppression rates [8, 9].

Studies also show that HIV‐related stigma amplifies existing discriminatory attitudes and practices in communities most affected by HIV. These include racism, sexism [9], homophobia and transphobia, as well as biased treatment of people who engage in sex work, people who use drugs and people who are incarcerated [10, 11]. U = U messaging can be wielded to advocate for removal of discriminatory policies and laws codifying racism, homophobia [12] and other biases, on the basis that discriminatory conditions block access to HIV treatment, care and prevention [13].

We know that thanks to treatment, PLHIV can lead longer, healthier lives – and U = U facts are aligned with HIV treatment goals. One large, international study found that PLHIV who were informed by their healthcare providers about U = U reported significantly more favourable health outcomes – including improved mental health, sexual health, overall health, medication adherence and rates of viral suppression compared to those who were not informed [14]. By discussing U = U with PLHIV, healthcare providers fulfil their ethical obligation to provide optimal care.

As advocates who have shared U = U with PLHIV for many years, we hear direct accounts of the joy, hope and freedom offered through U = U, such as these statements from PLHIV from all walks of life:

*“This breakthrough U = U science has changed my life…. There's hope.” Roscoe, USA*

*“I was always scared to bring another human being in the world with HIV until U = U empowered me. Because of U = U, I have two healthy baby boys.” Claire, Rwanda*

*“I can't wait for the people all around the world to be educated about U = U to end the stigma. I am ready to break free and live without fear of discrimination. Learning about U = U has given me so much to look forward to*.*” Victoria, Seychelles*

Improving the lives and health outcomes of PLHIV is the principal benefit of U = U. It is also a powerful tool for primary prevention. Based on these individual and public health benefits, tremendous progress has been made to support and institutionalize U = U, including endorsements by the World Health Organization, the U.S. President's Emergency Plan for AIDS Relief, the U.S. Centers for Disease Control and Prevention, the Joint United Nations Programme on HIV/AIDS and the United Nations. Many social, structural and legal barriers to optimal treatment are also barriers to prevention. Housing insecurity, unjust criminalization and lack of access to sexual and reproductive health services are just a few examples of systemic conditions that can lead to diminished viral suppression and thereby increase the likelihood of HIV transmission. Removing such barriers is an essential aspect of securing universal, robust access to HIV treatment. The U = U message underscores the relationship between these objectives and their role in reducing new HIV cases. U = U can thus be a key component in advocacy to achieve these goals – and advance justice for all PLHIV.

To strengthen the impact of U = U, we must widen its reach. Today, the evidence‐based U = U message is being communicated by a global movement of more than 1000 partners in 105 countries, including health ministries, research organizations and community‐based organizations [15]. Despite our progress in disseminating the value of U = U for personal and public health, it is not enough. Everyone has a role to play in disseminating this life‐saving message within and across their circles of influence, regardless of size (Table 1).

**Table 1:** A call to action for widespread adoption of U = U

Action	Primary responsible party
Prioritize U = U in global, regional and national HIV treatment, prevention and monitoring policies, guidelines and strategies	Global organizations and government entities
Educate healthcare providers and communities about the vital importance of U = U and how to communicate it to PLHIV, policymakers and the public	HIV/AIDS medical, certification and regulatory associations, and non‐governmental organizations
Advocate using U = U to remove social, structural and legal barriers to quality treatment, care and services	Advocacy organizations and activists
Raise awareness about U = U accurately, meaningfully and prominently in public health communications	HIV/AIDS information providers, including health ministries, non‐governmental organizations and private industry

Going forward, we need an unwavering focus on the needs of PLHIV. This means ensuring consistent access to treatment and services and removing barriers to individuals reaching an undetectable viral load. This is the best way to save lives, change public perceptions and prioritize the physical and emotional wellbeing of PLHIV – in short, the surest path to dismantling stigma and ending discrimination. On this Zero Discrimination Day, we see an enormous opportunity for clear, positive, evidence‐based communication about the value of U = U as a vehicle for fighting discrimination, advancing health equity for PLHIV everywhere and ending HIV epidemics globally.

## COMPETING INTERESTS

The authors have declared no competing interests.

## AUTHORS’ CONTRIBUTIONS

The authors collaboratively conceived the content of the paper.
